# Anti-GBM Disease in Pregnancy: Acute Renal Failure Resolved After Plasma Exchange, Hemodialysis, and Steroids

**DOI:** 10.1177/2324709615624232

**Published:** 2015-12-31

**Authors:** Mohammed Muqeet Adnan, Jordan Morton, Syed Hashmi, Sufyan Abdul Mujeeb, William Kern, Benjamin D. Cowley

**Affiliations:** 1 University of Oklahoma Health Sciences Center, Oklahoma City, OK, USA; 2 University of Illinois at Chicago, IL, USA

**Keywords:** antiglomerular basement membrane disease, pregnancy, acute kidney injury, glomerulonephritis, therapeutics

## Abstract

Antiglomerular basement membrane (GBM) disease presenting during pregnancy is uncommon. We present a case of a pregnant female who presented with acute renal failure requiring dialysis due to anti-GBM disease. She responded well to plasma exchange, high-dose steroids, and hemodialysis. Cyclophosphamide was discussed but not given at the patient’s request due to concerns for the well-being of the fetus. Unfortunately, she suffered a spontaneous abortion in her eighth week of pregnancy. Subsequently, she had progressive improvement in her renal function and became hemodialysis independent at 2 weeks after diagnosis. Her renal function returned to baseline 3 months after diagnosis. We present this case in detail and review the literature regarding anti-GBM disease in pregnancy.

A 17-year-old female, at 6 weeks of gestation, was evaluated for persistent nausea and vomiting thought due to the pregnancy. On admission to an outside hospital, the patient was found to have a temperature of 37.2°C, a hemoglobin 7.6 g/dL, and a serum creatinine of 6.20 mg/dL. Seven months prior, the patient’s hemoglobin was 11.8 g/dL and creatinine was 0.66 mg/dL. The patient was transferred to University of Oklahoma Medical Center for further evaluation of her acute renal failure. On transfer, temperature was 36.4°C, heart rate was 89 beats per minute, respiratory rate was 16 cycles per minute, and blood pressure was 147/61 mm Hg. Physical exam revealed obesity (body mass index = 43 kg/m^2^) but was otherwise unremarkable. Specifically there was no evidence of fluid overload. Laboratory findings were as follows: hemoglobin 6.51 g/dL, white blood cell count 10 300/mm^3^, platelet count 384 000/mm^3^, sodium 136 mEq/L, potassium 4.4 mEq/L, chloride 107 mEq/L, bicarbonate 21 mEq/L, blood urea nitrogen 26 mg/dL, and creatinine 6.47 mg/dL. Serum iron studies showed an iron of 25 µg/dL, total iron binding capacity of 185 µg/dL, iron saturation of 14%, and ferritin of 170 ng/mL. Urine analysis at admission showed pH of 6.5, specific gravity 1.009, 2+ protein, 3+ blood, and too numerous red blood cells (RBCs) to count. Urine glucose, ketones, bilirubin, and leukocyte esterase were all negative. Urine sediment was examined by the Nephrology Consult Team and was remarkable for too numerous to count RBCs and one RBC cast.

On the second day of hospitalization, the patient underwent a kidney biopsy in an effort to determine the etiology of her acute renal failure. Additional laboratory studies were also obtained including antiglomerular basement membrane (GBM) antibodies, complement C3 and C4 levels, antineutrophil antibodies, antineutrophil cytoplasmic antibodies, antiproteinase 3, anti-Smith, anti-double-stranded DNA, HIV antibody, hepatitis A IgM antibody, hepatitis B surface antigen, hepatitis B core IgM antibody, hepatitis C antibody, and antistreptolysin O. These test were pending at the time of the biopsy and were all subsequently reported as unremarkable except the anti-GBM antibody, which was elevated at 156 arbitrary units.

Hematoxylin and eosin–stained sections of the renal biopsy showed acute necrotizing and crescentic glomerulonephritis (Figure 1). There were no globally obsolescent glomeruli; however, there was moderate interstitial inflammation and mild interstitial fibrosis (Figure 2). Approximately half of the glomeruli had crescents, and approximately a third of the glomeruli had foci of necrosis. Immunofluorescence showed linear staining of the GBM for IgG consistent with anti-GBM disease (Figure 3).

**Figure 1.**
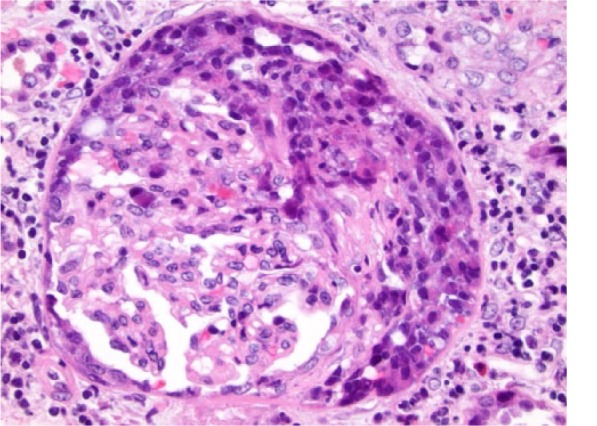
Hematoxylin and eosin–stained section of the renal biopsy showing crescentic glomerulonephritis with moderate interstitial inflammation and mild fibrosis with no evidence of vasculitis.

**Figure 2.**
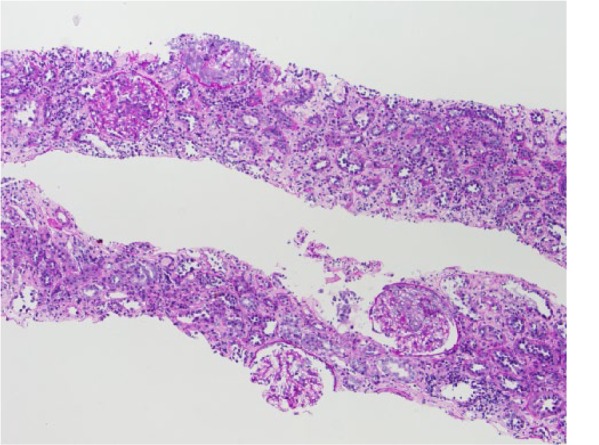
Low power (approximately 100×) hematoxylin and eosin–stained section of the renal biopsy showing crescentic glomeruli.

**Figure 3.**
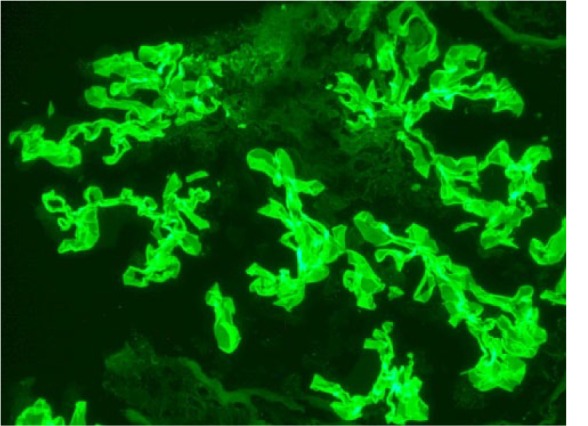
Immunofluorescence showing linear staining of the GBM staining for IgG.

Two units of packed red blood cells were administered with a posttransfusion hemoglobin of 8.9 g/dL. On hospital day 3, plasmapheresis was initiated. In addition, the patient was given intravenous methylprednisolone 1000 mg daily for 3 days, followed by oral prednisone 60 mg daily. The serum creatinine rose to 7.48 on day 5, and the patient developed oliguria, showing signs of fluid overload with pedal edema and lung crackles. Thus, hemodialysis was initiated.

The addition of cyclophosphamide was discussed at length with the patient and her family. In addition, Maternal-Fetal Medicine was consulted and emphasized that the health of the mother must be a priority. The patient was reluctant to initiate cyclophosphamide, since it was thought the risk of drug-induced abortion was high. Ultimately, it was decided to continue plasmapheresis and steroids and reevaluate the need for cyclophosphamide if the response was suboptimal. Unfortunately, on day 17 of hospitalization the patient suffered a spontaneous abortion. Post abortion, cyclophosphamide was not initiated at the patient’s request, due to concerns regarding future fertility. Thus, the patient was managed with hemodialysis, plasmapheresis, and steroids. Plasmapheresis began on day 5 of treatment, was performed daily with occasional breaks (Figure 4). Plasma was replaced with albumin, with plasma, or with a combination of albumin and plasma, based on fibrinogen levels and coagulation studies. Renal function recovered and dialysis was discontinued on day 14 of treatment. The patient was discharged 22 days after transfer. Plasmapheresis was continued intermittently after discharge, but travel became difficult for the patient and her family. In view of this, and with stable acceptable renal function and a significant fall in the anti-GBM antibody levels, plasmapheresis was discontinued on day 30 of treatment. Prednisone was tapered as an outpatient. Of interest, the patient became pregnant twice in the subsequent 18 months and again suffered spontaneous abortions early during these pregnancies. During the last pregnancy, serum creatinine was normal, anti-cardiolipin antibodies were negative, and anti-GBM antibodies were not present.

**Figure 4.**
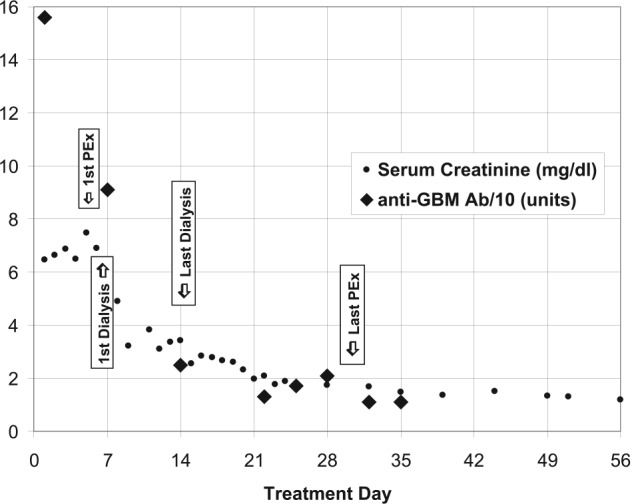
Serum creatinine and anti-GBM antibody/10 from time of transfer (PEx, plasma exchange).

## Discussion

Anti-GBM disease is an immune complex, small vessel vasculitis.^1^ The disease is characterized by the development of antibodies directed against an antigen intrinsic to the glomerular basement membrane and shared by the alveolar basement membrane. When these anti-GBM antibodies bind to the basement membrane, they activate the classical pathway of the complement system and start a neutrophilic inflammation that results in a crescentic glomerulonephritis.^1^ Anti-GBM disease, also called Goodpasture’s disease, generally produces a rapidly progressive glomerulonephritis that, despite modern treatment, results in renal failure or death in greater than two thirds of patients.^1^

Anti-GBM disease of pregnancy is uncommon. To date, 5 cases have been reported in the literature. Nilssen et al^2^ in their report of 4 cases described a pregnant patient who developed acute renal failure postpartum and never recovered renal function despite steroids, plasma exchange, and cyclophosphamide and remained dialysis-dependent. Yankowitz et al^3^ described a patient who had a diagnosis of Goodpasture’s disease, but with immunosuppressive therapy her anti-GBM levels became negative, and the patient had a successful delivery. Deubner et al^4^ described a case of anti-GBM disease that was diagnosed postpartum, and they proposed that the placenta might have been responsible for controlling the disease while the patient was pregnant. Al-Harbi et al^5^ described a 30-year-old female with rapidly progressive glomerulonephritis due to anti-GBM disease who needed dialysis during her pregnancy until delivery. Nair et al^6^ described a case of anti-GBM disease diagnosed during pregnancy that responded to plasma exchange and steroids. The pregnancy was terminated at 15 weeks, and post termination the patient’s renal function returned to normal with plasma exchange and steroids.^6^

We describe a pregnant female who was found to have acute renal failure during her sixth week of pregnancy. Based on the case reports, be it postpartum or ante partum, anti-GBM disease that presents during or after pregnancy is typically severe enough to cause oliguric renal failure. Treatment of anti-GBM disease causing rapidly progressing glomerulonephritis generally consists of plasma exchange, high-dose steroids, and cytotoxic agents such as cyclophosphamide. It is unusual to have renal recovery if kidney failure is severe enough to require dialysis to maintain electrolyte equilibrium and euvolemia.

We treated our patient with plasma exchange and high-dose steroids. Because the renal failure was worsening we started hemodialysis. Use of cyclophosphamide was discussed, but the patient declined its use due to concerns for the fetus. In spite of other aggressive measures, she suffered a spontaneous abortion. The patient also declined use of cyclophosphamide after the abortion due to concerns for future fertility. Plasma exchange was continued for more than a month (Figure 4). Her anti-GBM levels fell and were almost undetectable prior to discharge. Renal recovery gradually occurred and hemodialysis was ultimately discontinued (Figure 4). Steroids were gradually tapered, and kidney function returned to baseline. This case is unusual in that there was near complete renal recovery without cytotoxic therapy.

Pregnancy in general is a high-risk state, and exacerbation of autoimmune diseases is a common phenomenon during pregnancy. Though our patient developed rapidly progressive glomerulonephritis during pregnancy, it is unclear whether this was a triggering event, since she subsequently has been pregnant twice without evidence of recurrent anti-GBM disease or renal impairment. Nonetheless, it seems prudent that individuals who have been diagnosed with anti-GBM disease during pregnancy have close follow-up, especially during subsequent pregnancies, since recurrence of anti-GBM disease has been reported with subsequent pregnancies, and it has been suggested that resumption of immunosuppressive therapy may be needed.^3^
